# Predictive models in obstetrics: Lessons from the celestial *N*‐body problem

**DOI:** 10.1002/pmf2.70241

**Published:** 2026-01-19

**Authors:** Jeremy Boujenah, Patrick Rozenberg

**Affiliations:** ^1^ Department of Obstetrics and Gynecology Groupe Hospitalier Diaconesses Croix Saint‐Simon Paris France; ^2^ Department of Obstetrics and Gynecology Poissy‐Saint Germain Hospital, Poissy, France; ^3^ Paris Saclay University, UVSQ, Inserm, Team U1018, Clinical Epidemiology, CESP Montigny‐le‐ Bretonneux France

**Keywords:** *N*‐body problem, prediction: obstetrics, pregnancy

## 1 INTRODUCTION

The rise of personalized medicine, the growing autonomy of patients in therapeutic decision‐making, and increasing medico‐legal pressures have intensified the desire to “predict the future” and fueled the search for reliable predictive models.

In obstetrics, more than 100 models have been developed to predict outcomes such as preterm birth, fetal growth restriction, preeclampsia, or delivery outcomes. Yet, only a few have undergone proper external validation or demonstrated meaningful clinical impact [1]. Several factors may explain this limited predictive capacity: the multifactorial nature of obstetric outcomes, insufficient integration of biological and non‐biological interactions, unrecognized multilevel interdependencies, treatment paradoxes, or the rarity of outcomes (e.g., intrapartum hypoxic‐ischemic encephalopathy) relative to the number of predictors.

Most models rely on a priori variable selection or on univariate and multivariate analyses, often with questionable adjustment strategies. The dichotomization of continuous variables frequently leads to loss of information, and the inclusion of heterogeneous populations further challenges external validity [2]. Moreover, the relevance of studying perfectly representative populations has long been debated.

In the United States, for instance, the persistent difficulty in reducing cesarean delivery rates has been attributed by some authors to geographic, demographic, and social factors that current predictive models cannot adequately account for, despite updated clinical guidelines on labor management [3]. Likewise, recent debates surrounding new labor curves and their questionable internal and external validity suggest that our limited and static understanding of pregnancy itself may underlie the difficulty of prediction in obstetrics.

Understanding why predictive models perform poorly and are difficult to implement in clinical practice requires revisiting the very nature of the phenomena they aim to capture. This calls for combining an ontological approach (acknowledging that unobserved elements of reality may explain observed outcomes) with an epistemological one (recognizing that assumed causes may not, in fact, be the true causes).

The challenge of prediction is not unique to medicine. Physics, through the study of celestial mechanics, has faced similar questions: how systems governed by clear physical laws can nonetheless exhibit unpredictable, chaotic behavior?

In this perspective, we propose to draw an analogy between pregnancy and a celestial *N*‐body system—a classical problem in celestial mechanics known for its deterministic chaos and sensitivity to initial conditions. This analogy offers a new conceptual framework to understand the inherent unpredictability of obstetric outcomes and the limitations of current predictive approaches.

## 2 THE CELESTIAL MECHANICS OF THE *N*‐BODY PROBLEM: DETERMINISTIC CHAOS

In physics, a system refers to a set of interacting components whose evolution over time is governed by well‐defined laws. The state of a system at a given moment is described by a collection of variables—such as position, mass, and velocity—that summarize all the information required to determine its future evolution under those laws.

Determinism implies that if the initial conditions of a system are known exactly, its future evolution can, in principle, be fully determined by the laws of gravitational physics. *Chaos*, however, refers to the phenomenon whereby minute differences in initial conditions can lead to disproportionately large divergences in long‐term behavior, thereby limiting practical predictability despite the absence of randomness.

In celestial mechanics, the motion of astronomical bodies illustrates this principle. A two‐body system (e.g., the Earth orbiting the Sun) remains predictable over long timescales.

By contrast, the introduction of a third gravitationally interacting body (e.g., the Alpha Centauri system) gives rise to what is known as the three‐body problem in which the system becomes highly sensitive to initial conditions. In this setting, infinitesimal variations in initial parameters may result in qualitatively different trajectories, that is, different possible time courses of the system's evolution.

Importantly, these bodies do not interact through direct contact but through a gravitational field (defined by their respective masses, distances, and the geometry of space‐time). Although this field is not a material object, it fully determines how trajectories evolve. So, such a system is inherently complex and nonlinear. The resulting dynamics is that small changes in one component can produce disproportionately large effects on the overall system.

This apparent unpredictability does not stem from a lack of data but from the intrinsic instability of the system's dynamics. Even in a universe governed by fixed physical laws, the unpredictable evolution of a system demonstrates that determinism does not ensure predictability [4]. In this general three‐body problem—that is, three gravitationally coupled bodies—it is not possible to derive a single exact analytical solution describing the full long‐term trajectory of the system, even though the governing equations are known. An analytical solution refers to a closed‐form mathematical expression that describes the system's evolution exactly, without iterative computation. In systems involving three or more interacting components, predictability is limited not by ignorance of the laws but by the intrinsic instability of the system's dynamics. As Poincaré noted, knowing the governing equations does not guarantee the ability to explicitly determine the solution, that is, the system's future evolution.

An analytical solution has been found only for specific configurations of the *N*‐body problem, such as the restricted three‐body problem in which one body has negligible mass. In all other cases, numerical approaches provide approximate solutions by iteratively estimating a range of possible trajectories, each subject to quantifiable errors and uncertainty [5]. In substance, numerical approaches estimate the system's evolution step by step, producing approximate trajectories whose uncertainty can be quantified and propagated over time. This ensemble of solution refers to a set of possible trajectories that are all compatible with the same initial information

In sum, beyond the absence of a general analytical solution, the historical development of the *N*‐body problem offers a key conceptual lesson for the prediction in obstetrics:

‐ Even when governing low are well understood, prediction in complex systems has progressively shifted from the search for exact expressions toward the acceptance of approximate, local, and time‐limited solutions.
‐ Rather than attempting to eliminate uncertainty, modern approaches explicitly represent it through ensembles of possible paths, numerical simulations, and probabilistic bounds.

This perspective provides a natural framework for understanding pregnancy and childbirth as complex dynamical processes involving multiple interacting agents.

## 3 PREGNANCY AND CHILDBIRTH: A METAPHOR FOR A THREE‐BODY PROBLEM (FIGURE 1)

Maternal characteristics—including anthropometric, biological, genetic, socioeconomic, and environmental factors, as well as medical history and uterocervical dynamics—interact continuously with fetal characteristics such as genetic profile, growth, stress adaptation, and placental function. To these must be added the features of the medical system itself: the type of maternity unit, day or night workload, decision thresholds, adherence to protocols, team reactivity, and the individual dimension of the clinician. All these elements interact constantly, particularly during labor and delivery.

**FIGURE 1:**
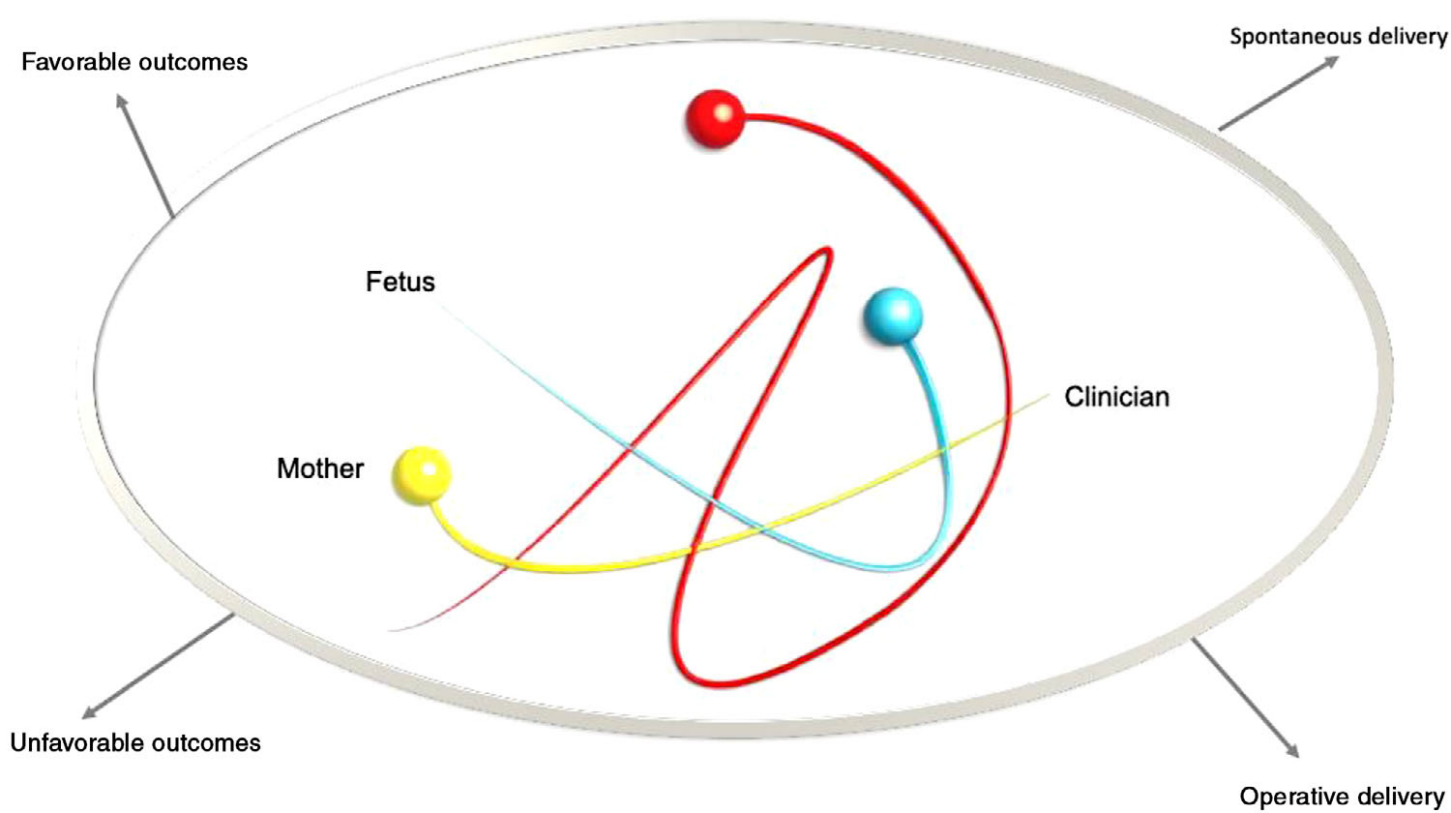
Illustration of three divergent trajectories intersecting and influencing one another.

Medical decisions, in turn, modify the natural course of labor and are themselves secondary to the ongoing maternal and fetal dynamics. The outcome of childbirth thus represents a complex, multifactorial phenomenon—what Kenneth Rothman described as a web of causation [6].

Labor can be viewed as the deterministic outcome of pregnancy: once the system (the pregnancy) reaches a given state (the onset of labor), its evolution inevitably leads to delivery. In theory, if all the initial conditions—maternal uterine environment, fetal state, and clinician‐related factors—were known with perfect accuracy, the future course of the system could be predicted.

Moreover, by analogy with *N*‐body problem, interactions within the obstetric system are mediated by a dynamic “field” composed of biological forces (uterine activity, placental function, and fetal adaptation), decision‐making forces (clinical judgment, protocols, and technological availability), and social forces (institutional norms, medico‐legal context, social position, and access to care). These forces do not exist as isolated predictors but as relational structures that continuously shape interactions between the mother, fetus, and clinician.

In practice, however, perfect knowledge of these conditions is unattainable. Even minimal variations between two apparently similar pregnancies can result in entirely different outcomes. This sensitivity to initial conditions embodies what might be called obstetrical chaos—a deterministic system whose evolution remains unpredictable.

In concrete terms, this unpredictability concerns the temporal course of labor rather than its inevitability. Labor can be described as a trajectory, defined as the succession of clinical states through which the mother–fetus–clinician system evolves over time. Similar initial conditions—such as comparable cervical dilation and maternal‐fetal status at onset—may nonetheless give rise to divergent trajectories over the following hours, with different patterns of progression and intervention.

## 4 IMPLICATIONS OF THE *N*‐BODY ANALOGY FOR PREDICTIVE MODELS IN OBSTETRICS

Current obstetric research oscillates between three modeling paradigms—descriptive, predictive, and causal (Table 1). While descriptive studies help us understand population patterns, and causal models aim to identify levers for intervention, most clinical tools belong to the predictive domain. Yet, applying predictive models to a system as dynamic and interdependent as the mother–fetus–clinician triad risks oversimplifying a reality governed by feedback loops and sensitive initial conditions—the very hallmarks of deterministic chaos.

**TABLE 1:** Conceptual comparisons of the statistical models.

Aspect	Descriptive model	Predictive model	Causal model
Goal	Describe associations and distributions.	Estimate the probability of a future outcome.	Identify and quantify cause–effect relationships.
Typical question	“What is the relationship between X and Y?”	“What is the risk that Y occurs if X is observed?”	“Does X cause Y, all else being equal?”
Interpretation	“X and Y are associated in this population.”	X is a predictive marker of Y.”	“X causally affects Y after adjustment for Z.”
Variable selection	Based on clinical interest or statistical significance (*p* < 0.05); often exploratory.	Based on predictive performance (AUC, *R* ^2^); methods include LASSO, Random Forest.	Based on causal graphs (DAGs): adjusting for confounders, excluding mediators or colliders.
Statistical methods	Simple/multiple regression, association tests (*χ* ^2^, *t*‐test).	Logistic regression, machine learning (XGBoost, neural networks), cross‐validation.	Adjusted regression, propensity scores, instrumental variables, structural marginal models.
Data type	Descriptive or observational data.	Observational or large‐scale data (registries).	Observational or experimental data (RCTs, quasi‐experiments).
Use in obstetrics	Characterize maternal‐fetal factors or labor patterns.	Predict cesarean delivery, preterm birth, or complications.	Estimate the effect of an intervention or exposure.
Strength	Improves understanding of population trends.	Supports risk stratification and patient counseling.	Guides clinical or policy decisions.
Limitations	No causal inference; may overlook dynamic interactions.	Sensitive to unmeasured or chaotic factors.	Requires strong, often unverifiable assumptions. Generalizability of RCT (efficacy is demonstrated rather than effectiveness).

Predictive models in obstetrics are often based on linear regressions or simplified Bayesian frameworks and attempt to forecast events such as the timing or mode of delivery, fetal weight, or specific risks—as if the system were stationary and isolated. Obstetric systems display continuous feedback with trajectories that are neither independent nor linear. Moreover, several determining factors are either unmeasured or inherently unmeasurable—for instance, clinical intuition, team fatigue, or the psychological experience of the patient. Moreover, predictive models often treat social characteristics, such as race, as fixed predictors, while they in fact reflect dynamic and relational forces shaped by institutional practices and access to care. By freezing these forces into stable variables, models risk misrepresenting interactions that unfold through largely unobservable fields, thereby reinforcing the intrinsic limits of prediction in obstetrics. This dynamic has been empirically illustrated in studies of VBAC prediction tools, which show how the use of race as a predictor may transform socially mediated differences into seemingly intrinsic properties of individuals [7].

The initial conditions of both the mother and the fetus (such as placental reserve or fetal adaptation to hypoxia) are imperfectly known. As in the three‐body problem, this system can abruptly shift phase—for example, when an emergency cesarean is required for cord prolapse or placental abruption. This sensitivity to initial conditions—a key feature of chaos theory—illustrates why small differences can lead to vastly divergent clinical outcomes.

In other words, predictive models in obstetrics carry a deterministic ambition in a fundamentally chaotic world. Wynants et al. identified several methodological pitfalls in the development of prediction models, some of which question their very applicability to obstetrics [8]:

‐ The inclusion of numerous and nonlinear interactions.
‐ The statistical imputation of missing data in inherently variable biological systems.
‐ The prediction of rare outcomes.

In such situations, the authors suggested either constructing simpler models or, in some cases, refraining from modeling altogether.

Labor provides a particularly instructive example. The three interacting agents—the mother, the fetus, and the clinician—whose continuous interactions and feedback render the global trajectory of the process inherently unpredictable. In this context, prediction is not about foreseeing the full course of labor, but about recognizing when and how to intervene to keep the system within a viable dynamical regime. This perspective implies a shift in the predictive question itself. Rather than asking the intrinsically chaotic and clinically unanswerable question “How long will labor last and how will it end?,” predictive models should address localized, time‐bounded questions such as: What is the probability of satisfactory progression during the next hours of the latent phase (or another phase)? Sequential Bayesian predictive models in obstetrics formalize the same epistemological shift that occurred in celestial mechanics: they do not aim to recover an exact trajectory but to maintain an updated distribution over plausible system states, thereby supporting timely and informed interventions in the presence of intrinsic instability (Table S1).

In fact, celestial mechanics has taught us to think of prediction as a continuous updating of plausible beliefs. Sequential Bayesian models in obstetrics provide the clinical mathematical translation of this approach. In clinical practice, the output of such a model could be expressed as: “Given the current state, there is an 80% probability of progressing toward a favorable outcome over the next two hours.”

Fortunately, some situations can be easier. Similarly, as the three‐body problem in celestial mechanics can be simplified when one body's mass is negligible, certain obstetric outcomes may become predictable when one of the three agents—mother, fetus, or clinician—plays a passive or secondary role:

‐ Predicting the outcome of a trial of labor in the case of severe fetal growth restriction with abnormal Doppler findings and breech presentation at 41 weeks, where the fetus's compromised condition largely dictates the result.
‐ Predicting the outcome of a breech trial of labor when strict institutional protocols limit clinical discretion, thereby reducing variability in clinician behavior.

In fact, in these situations, the utility of predicting these phenomena whereby one of the factors would ultimately dictate the outcome of the labor is doubtful.

From a statistical perspective, both celestial mechanics and obstetrics, the solutions are constrained by how error is quantified and managed. In chaotic dynamics, uncertainty is deterministic and exponential—that is, it grows with variability and incomplete knowledge of initial conditions. In biostatistics, uncertainty is considered random in an ideal model, or systematic (bias) when linked to data imperfections, and is expressed through confidence intervals.

While predictive model performance is often summarized by a receiver operating characteristic (ROC) curve and its confidence interval, individual‐level predictions typically yield a single risk estimate without an associated confidence interval—despite the fact that such uncertainty could, in principle, be computed using more complex methods. The ongoing debate surrounding the terminology of this interval—whether to call it “confidence,” “compatibility,” or “uncertainty” [9]—reflects our persistent difficulty in obtaining and interpreting estimates within a system as complex as pregnancy.

**FIGURE 2:**
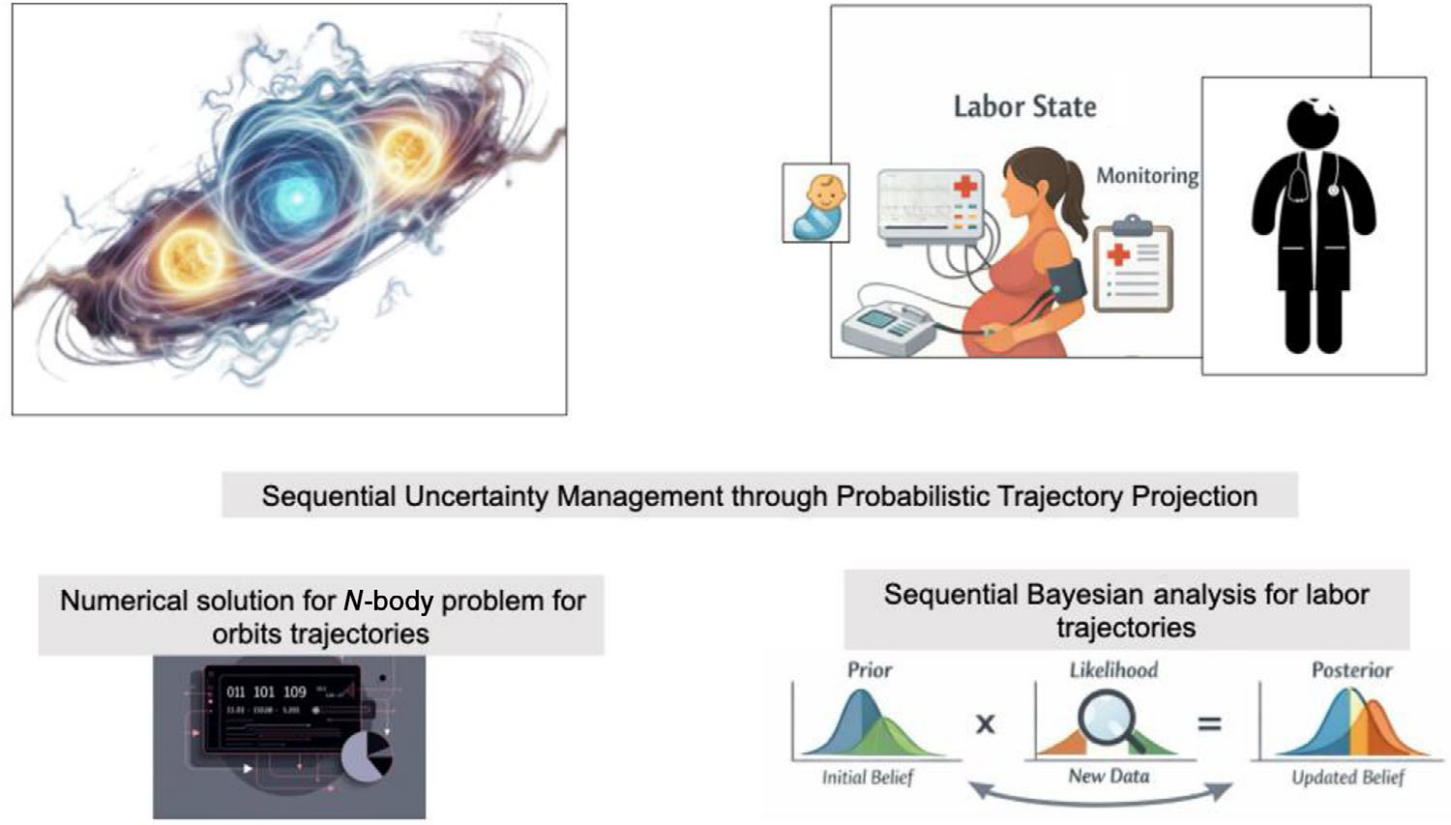
Parallel between orbital and labor trajectories: managing the unknown using probabilistic projection.

In sum, rather than aiming for universal or exact predictive expressions, models should be conceived as local approximations, valid within constrained clinical contexts and limited time horizons. Insights from celestial mechanics show that prediction is no longer absolute, but structured, bounded, and probabilistic. The value of such models lies less in precise point estimates than in their ability to delineate ranges of plausible trajectories, guide clinical vigilance, and support—rather than replace—human judgment under intrinsic uncertainty (Figure 2).

## 5 CONCLUSION

This cosmic parallel is not a rejection of predictive modeling in obstetrics, nor a claim that pregnancy is universally chaotic or unpredictable. Rather, it is a transdisciplinary reflection—an invitation to caution in the confidence we place in predictive models, particularly as their number and sophistication are rapidly increasing in the era of artificial intelligence and big data.

Recognizing the obstetric system as a dynamic, interdependent, and potentially chaotic network may ultimately lead to more realistic models—ones that do not seek to eliminate uncertainty, but to understand and integrate it as an intrinsic property of life itself.

## CONFLICT OF INTEREST STATEMENT

The authors declare no conflicts of interest.

## FUNDING INFORMATION

The authors received no specific funding for this work.

## Supporting information



Supporting information
