# Supplementary material for: Predictive models in obstetrics: Lessons from the celestial N‐body problem
Source: Pregnancy (Hoboken). 2026 Jan 19;2(1):e70241. doi: 10.1002/pmf2.70241 (PMC13344523; doi:10.1002/pmf2.70241)
Supplement: Supplementary file 1 [file PMF2-2-e70241-s001.docx]

| **Concept** | **Celestial Mechanics** | **Obstetrics** | **Sequential Bayesian model** |
| --- | --- | --- | --- |
| System state | Orbital state (position, mass velocity) | Labor state (cervical dilation, contractions, fetal and maternal status), clinician’s practice | **Prior information**: initial belief about the system state |
| Observations | Successive measurements of positions/velocities | Clinical monitoring: cervical exams, contractions, fetal heart rate | **Data / likelihood**: probability of observing the data given the system state |
| Ensemble of trajectories | Ensemble of possible orbits | Ensemble of possible labor trajectories | **Posterior distribution**: updated beliefs combining prior and likelihood |
| Sequential update | Numerical integration step by step | Updating with each new observation | **Posterior becomes new prior** for the next observation; sequential updating of beliefs |
| Key feature | Deterministic simulation with sensitivity to initial conditions | Labor evolution sensitive to initial state and interventions | **Sequential inference**: continuous updating, not a single exact prediction |

**Supplementary Table : Conceptual parallel between N-body simulations in celestial mechanics and sequential Bayesian models in obstetrics**.

*In both cases, the system’s state evolves over time, and uncertainty is managed step by step. In celestial mechanics, numerical integration produces an ensemble of possible trajectories starting from slightly different initial conditions.*

*In obstetrics, sequential Bayesian inference updates the distribution of plausible labor trajectories as new data become available, combining prior beliefs with the likelihood of observations to produce a posterior that informs the next update.*

*In this way, both approaches emphasize sequential updating of plausible states rather than attempting to predict a single exact outcome*
